# Applications of Environmental Epidemiology in Addressing Public Health Challenges in East Asia

**DOI:** 10.1155/2015/462426

**Published:** 2015-07-30

**Authors:** Shih-Bin Su, Jamal H. Hashim, Chong-huai Yan

**Affiliations:** ^1^Department of Occupational Medicine, Chi Mei Medical Center, Tainan, Taiwan; ^2^Southern Taiwan University of Science and Technology, Tainan, Taiwan; ^3^United Nations University-International Institute for Global Health, UKM Medical Centre, 56000 Kuala Lumpur, Malaysia; ^4^MOE-Shanghai Key Laboratory of Children's Environmental Health Xinhua Hospital Affiliated to Shanghai Jiao Tong University School of Medicine, China

The East Asian region is a vibrant and challenging part of the world when it comes to public health issues, with its diverse economies, ethnicity, cultures, and geographical and geological features. It covers about 12 million km^2^ or about 28% of the Asian continent and is about 15% bigger than Europe. More than 1.5 billion people or about 38% of the Asian population live in East Asia. The region is one of the world's most populated places, with a population density of 133 inhabitants per km^2^. While communicable diseases still plague the less developed nations of East Asia, noncommunicable diseases are predominant in the more developed nations and are also making their presence in the developing nations. Besides being threatened by environmental pollution from its rapid pace of urbanization and industrialization, it is also an area prone to natural disasters like volcanic eruptions, earthquakes, tsunamis, typhoons, droughts, and floods. Therefore, not only human lives are at stake, but also the ecosystems, natural resources, and properties. These issues are multidisciplinary in nature and require a multipronged approach. Environmental epidemiology is such an approach that assesses and evaluates a public health problem from various angles and perspectives to derive a scientifically sound decision-making process. The outcomes of good environmental epidemiology should feed into sound risk management and risk communication practices for the protection of human health and safety.

The accepted papers of this special issue comprise critical and new areas of research and recent advances on challenging issues in different fields of environmental epidemiology in addressing public health challenges in East Asia.

In the study by C.-J. Peng et al., the authors used food balance data to compare quality, quantity, and trends of food supply from 1984 to 2009 and the degree of food westernization in Taiwan with other Asian countries and the world continents. They concluded that Taiwan's food supply provided sufficient quantity in food energy, with the lowest cereals/roots supply and rice to wheat ratio, but the highest meat and oil supplies per capita among the 10 studied Asian countries. Taiwan's food supply showed the most westernization among these countries.

T.-Y. Yang et al. conducted a case-control study consisting of 70 pathologically confirmed skin cancer patients and 210 age-gender-matched participants with genotyping of 12 selected polymorphisms. Individuals carrying three risk polymorphisms of EPHX1 Tyr113His, XPD C156A, and any one risk polymorphism of the GSTs presented a 400% increased risk of arsenic-induced skin cancers compared to those with less or equal to one polymorphism. They concluded that GSTs, EPHX1, and XPD are potential genetic factors for arsenic-induced skin cancers.

C.-K. Lin et al. reviewed the data from Taiwan Cancer Registry to determine the standardised incidence ratio (SIR) of malignant pleural mesothelioma (MPM) in workers exposed to asbestos in Taiwan. They found that the highest risk of MPM was among male asbestos workers employed before 1979 and working for more than 20 years in asbestos-related factories.

C.-C. D. Lee et al. used the general additive model (GAM) to understand the association between hand-foot-mouth disease (HFMD) and latitude, as well as meteorological factors for islands in East Asia, namely, Japan, Taiwan, Hong Kong, and Singapore, from 2012 to 2014. They concluded that weather conditions and geographic location could play some role in affecting HFMD epidemics. Regional integrated surveillance of HFMD in East Asia is needed for mitigating the disease risk.

Y.-F. Liu et al. collected a total of 177 consecutive patients who underwent the outpatient treatments at departments of cardiology. They were prospectively enrolled to investigate whether neck circumference (NC) is associated with factors of chronic kidney disease (CKD) among patients with cardiometabolic risk. They concluded that NC is associated with cardiovascular metabolic risks and can be measured easily and routinely in the future.

C.-H. Chiang et al. enrolled participants who were 50 years old and older from Taiwan's National Health Insurance Research Database. Participants were recruited from 2000 to 2004 and then followed up until death or the end of 2011. They found the incidence of dementia was significantly higher in the LUTS[+] group than in the LUTS[−] group (124.76 versus 77.59/1000 person-years). The authors concluded that LUTS are associated with increased risk of subsequent dementia among the elderly population.

